# Assessing the Role of Post-Translational Modifications of Mitochondrial RNA Polymerase

**DOI:** 10.3390/ijms242216050

**Published:** 2023-11-07

**Authors:** Karlie R. Platz, Emma J. Rudisel, Katelynn V. Paluch, Taylor R. Laurin, Kristin E. Dittenhafer-Reed

**Affiliations:** Department of Chemistry and Biochemistry, Hope College, Holland, MI 49423, USA

**Keywords:** post-translational protein modification, acetylation, phosphorylation, mitochondrial DNA, mitochondrial genome, mitochondrial proteins, transcription

## Abstract

The mitochondrial proteome is subject to abundant post-translational modifications, including lysine acetylation and phosphorylation of serine, threonine, and tyrosine. The biological function of the majority of these protein modifications is unknown. Proteins required for the transcription and translation of mitochondrial DNA (mtDNA) are subject to modification. This suggests that reversible post-translational modifications may serve as a regulatory mechanism for mitochondrial gene transcription, akin to mechanisms controlling nuclear gene expression. We set out to determine whether acetylation or phosphorylation controls the function of mitochondrial RNA polymerase (POLRMT). Mass spectrometry was used to identify post-translational modifications on POLRMT. We analyzed three POLRMT modification sites (lysine 402, threonine 315, threonine 993) found in distinct structural regions. Amino acid point mutants that mimic the modified and unmodified forms of POLRMT were employed to measure the effect of acetylation or phosphorylation on the promoter binding ability of POLRMT in vitro. We found a slight decrease in binding affinity for the phosphomimic at threonine 315. We did not identify large changes in viability, mtDNA content, or mitochondrial transcript level upon overexpression of POLRMT modification mimics in HeLa cells. Our results suggest minimal biological impact of the POLRMT post-translational modifications studied in our system.

## 1. Introduction

Mitochondria are organelles that perform essential functions in energy production and cellular metabolism. Mitochondria contain their own DNA (mtDNA), which encodes 13 subunits necessary for oxidative phosphorylation. Over 1500 other mitochondrial proteins, including the 77 remaining oxidative phosphorylation subunits, and the machinery required for transcription and translation of mtDNA, are encoded by the nuclear genome. Thus, transcription of the nuclear and mitochondrial genomes must respond in concert to meet changing energetic needs of the cell. Mitochondrial transcription is initiated by mitochondrial transcription factor A (TFAM), which forms a complex with mitochondrial RNA polymerase (POLRMT) [1,2]. This complex recruits mitochondrial transcription factor B2 (TFB2M) to assist in promoter melting, which allows POLRMT to initiate transcription [3]. While POLRMT is mainly known as the RNA polymerase, it is also required to synthesize the RNA primer necessary to initiate lagging strand synthesis during mtDNA replication [4]. Detailed protein structures exist that have allowed for elucidation of the mechanism of mitochondrial transcription [5,6]; however, there is a need to more deeply understand how the mtDNA transcriptional machinery is controlled to respond to the cell’s energy demands.

Reversible protein post-translational modifications (PTMs), including lysine acetylation and phosphorylation of serine, threonine, and tyrosine, are one mechanism regulating protein function in the cell. PTMs are widespread in the mitochondrial proteome, including on the mitochondrial transcription machinery [7,8,9,10,11]. While PTMs are abundant in the mitochondrial proteome, their biological function remains largely uncharacterized. We set out to determine whether PTMs of the mitochondrial transcriptional machinery regulate mitochondrial gene expression to adapt to the energetic needs of the cell, akin to mechanisms controlling nuclear gene expression via DNA-binding proteins [12]. In this study, we focus on the role of site-specific acetylation and phosphorylation in the regulation of POLRMT. 

POLRMT is the only RNA polymerase residing in the mitochondria, making it essential for oxidative phosphorylation and cell viability [13]. POLRMT is capable of sensing the metabolic state of the cell and regulating the expression of some mitochondrial RNAs [14], but other regulatory mechanisms of POLRMT function remain unknown. Previous work also identified point mutations of the *POLRMT* gene with profound impacts on human health. A broad range of physical and neurological effects were observed in an eight-patient cohort with *POLRMT* mutations [15]. Analysis of these variants using an in vitro model found decreased mitochondrial transcript levels and replication primer synthesis. The drastic effect of these point mutations suggests that the modifications induced by site-specific acetylation and phosphorylation may also impact POLRMT function and serve as a means of transcriptional regulation. Further, elucidating the mechanisms regulating POLRMT and impacting mtDNA transcription may advance the understanding of human mitochondrial diseases. In this study, we describe the use of POLRMT point mutants designed to mimic acetylation (lysine to glutamine, K→Q) or phosphorylation (threonine to glutamate, T→E) at three structurally distinct regions in POLRMT. We monitor the binding affinity of POLRMT modification mimics to the light strand promoter of mtDNA, We assessed cell viability, mtDNA content, and mitochondrial transcript levels in HeLa cells overexpressing POLRMT modification mimics. 

## 2. Results

### 2.1. POLRMT Post-Translational Modifications Mapped by High-Resolution Mass Spectrometry

**Figure 1:**
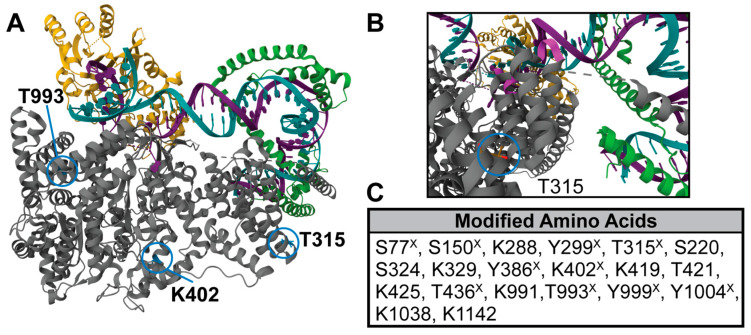
Structure of POLRMT and location of PTM sites analyzed. (**A**) Structure of the human mitochondrial initiation complex (PDB: 6ERP) [2]. POLRMT (gray) pictured in complex with transcription factor A (TFAM, green) and transcription factor B (TFB2M, yellow) at the light strand promoter (non-template strand, purple; template strand, teal). PTM sites analyzed in this study are circled in blue. (**B**) A zoomed in view of the structural region containing T315 and the light strand promoter.(**C**) A list of all acetylated and phosphorylated amino acids in POLRMT found in prior work [11] (marked with ^X^) and our study by mass spectrometry in human cell lines. Our work obtained 21% sequence coverage of POLRMT and identified seven acetylated lysines and three phosphorylation sites (Supplementary Table S1).

Previous high-throughput proteomics studies identified acetylation (K402), succinylation (K622), ubiquitination (K394, K532, K1197, K1205, K1213), and phosphorylation sites (S77, S150, Y299, T315, Y386, T436, T993, Y999, Y1004) on POLRMT in a variety of human cell lines [11,16]. We used high-resolution liquid chromatography tandem mass spectrometry (LC-MS/MS) to map additional PTMs on POLRMT. POLRMT was overexpressed in HeLa cells, immunoprecipitated, subject to digestion with trypsin or Glu-C, and analyzed by LC-MS/MS. Across two biological replicates, we obtained 21% sequence coverage for POLRMT and identified seven acetyl sites (K288, K329, K419, K425, K991, K1038, K1142) and three phosphorylation sites (S220, S324, T421) (Figure 1 and Supplementary Table S1). Interestingly, we did not find any modifications previously identified [11,16]. Improved sequence coverage would increase the number of identified PTMs. HeLa cells have been used in previous proteomic studies of mitochondrial proteins [17], analysis of mitochondrial gene expression and oxidative phosphorylation [18,19], and studies of POLRMT [20,21]. We chose to use HeLa cells in our study for these reasons and their amenability to transfection and manipulation. Collectively, proteomics analyses show that POLRMT is subject to numerous modifications; however, the biological function of these modifications is unknown. PTMs of human and yeast nuclear RNA polymerase have previously been shown to regulate polymerase function [22,23,24], suggesting the possibility for a similar mechanism in mitochondria.

### 2.2. Acetyl and Phosphomimics Used to Monitor Changes in Promoter Binding by Fluorescence Polarization

Site-directed mutagenesis was employed to create acetyl and phosphomimics of POLRMT. Acetylated lysines were mimicked by mutating lysine to glutamine (K→R), and phosphorylated threonine was mimicked by mutating threonine to glutamate (T→E). We chose to create an acetyl mimic of lysine 402 (K402) and phosphomimics of threonines 315 and 993 (T315, T993). This allowed for the study of PTMs in three structurally and functionally distinct regions of POLRMT. 

POLRMT is comprised of four domains: the N-terminal extension, the pentatricopeptide repeat domain (PPR), the N-terminal domain, and the C-terminal domain (Figure 1) [6]. Lysine 402 is found in the N-terminal domain of POLRMT, which contains structural elements that are homologous to the promoter binding/recognition structures found in the T7 phage RNA polymerase and may be a prime region for regulation [25]. Threonine 315 is located in the PPR domain of the N-terminal extension. The PPR domain is important for interactions between POLRMT and the DNA backbone upstream of the transcription start site; however, T315 is not one of the previously mapped amino acids that form contacts with DNA (amino acids 220–225, 252–255) [2]. Additionally, interactions between TFAM and the N-terminal extension domain, which includes the PPR domain, serve to anchor POLRMT to mtDNA at the promoter during transcription initiation, making PTMs to this region of the protein interesting from a regulatory standpoint [2]. Threonine 993 is part of the finger sub-domain of the C-terminal domain of POLRMT. The palm and fingers subdomains comprise the catalytic subunit of POLRMT and make contacts with DNA downstream of the transcription start site [5]. T993 lies within the O-helix of the finger subdomain, a highly conserved *α*-helix composed of residues 986–1000 required for nucleotide triphosphate binding. Phosphorylation of threonine 993 is of special interest due to its proximity to lysine 991, an important residue in the O-helix. Binding of a sulfate ion to lysine 991 is thought to stabilize incoming nucleotide triphosphates during transcription elongation, suggesting an impact of the addition of a negatively charged phosphate at this location [26]. Threonine 993 is the only amino acid conserved from yeast to humans. 

**Figure 2:**
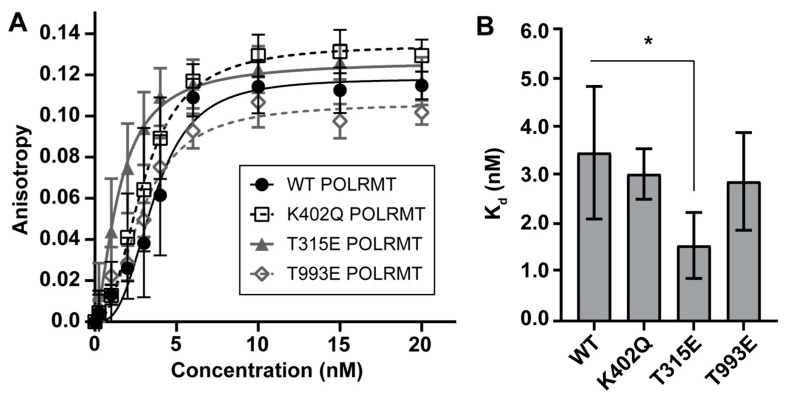
Binding affinity of POLRMT PTM mimics for the light strand promoter determined by fluorescence polarization. A slight enhancement of promoter-binding affinity was observed for the phosphomimic at T315. (**A**) 5′-fluorescein labeled light strand promoter duplex DNA (−40 to +10, 3 nM) was titrated with increasing concentrations of POLRMT WT or PTM mimic. Anisotropy values from several independent experiments were averaged and plotted. The average binding data were fit using specific binding with the Hill slope to determine the dissociation constants (K_d_) shown in (**B**). Error bars represent the standard error of the curve fit for a single trial. (**B**) Binding constants are unchanged between WT (3.45 ± 0.76 nM), K402Q (3.02 ± 1.02 nM), and T993E POLRMT (2.87 ± 0.17 nM). Binding affinity is increased by approximately two-fold for T315E POLRMT (1.55 ± 0.78 nM) when compared to WT (* indicates significance, *p* = 0.002, Student’s *t*-Test). The data represent the average and standard deviation of binding constants calculated from 4–6 trials for each mutant.

Wild type (WT), K402Q, T315E, or T993E POLRMT was recombinantly expressed and purified. All purified proteins lacked the mitochondrial targeting sequence and were expressed with a polyhistidine tag on the N-terminus. The human POLRMT proteins expressed in *E. coli* should have minimal to no post-translational modifications. Fluorescence polarization was used to study the effect of POLRMT modifications on binding to the light strand promoter of mtDNA (region −41 to +10). The K_d_ for wild-type (WT) POLRMT binding to the light strand promoter was 3.45 ± 0.76 nM (N = 7). This binding affinity is comparable to previous reports [27]. No significant differences in promoter binding affinity were observed for the K402 acetyl mimic (K402Q, K_d_ = 3.02 ± 1.02 nm; N = 5) or the T993 phosphomimic (T993E, K_d_ = 2.87 ± 0.17 nM, N = 4) (Figure 2). A slight decrease in K_d_ when compared to WT POLRMT was observed for the T315 phosphomimic (T315E, K_d_ = 1.55 ± 0.78 nM, N = 5). While the difference in binding affinity between T315E and WT POLRMT is statistically significant (*p* = 0.002, two-sided *t*-test), the biological relevance of such a small change in K_d_ of less than 2 nM between the WT and T315E POLRMT is unclear. 

### 2.3. The Impact of Overexpression of the POLRMT T315 Phosphomimic in HeLa Cells on Cell Viability, mtDNA Content, and Mitochondrial Transcription

**Figure 3:**
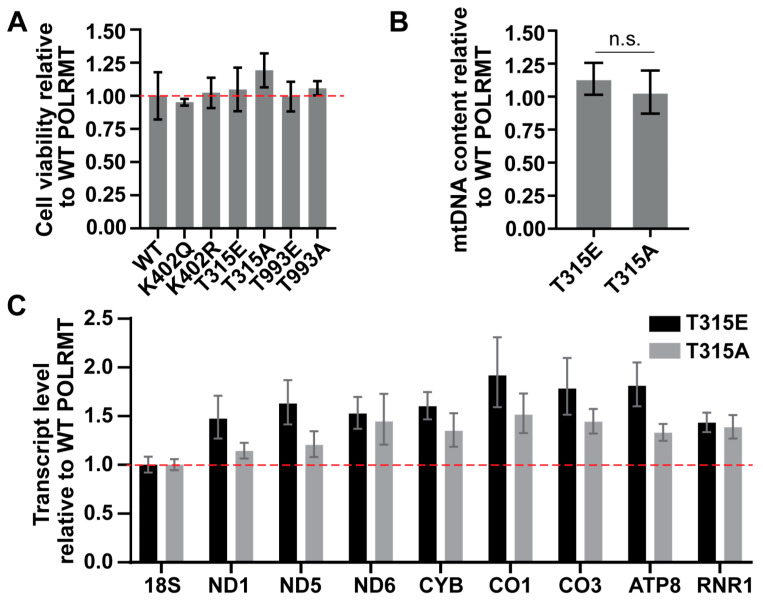
The effect of the overexpression of POLRMT PTM mimics on viability, mtDNA content, and mitochondrial transcript levels in HeLa cells. (**A**) Overexpression of POLRMT modification mimics does not alter cell viability. An alamarBlue^TM^ assay was used to monitor cell viability of HeLa cells transfected with POLRMT modification mimics after 48 h. Cell viability relative to the overexpression of WT POLRMT is presented. The average and standard deviation of five technical replicates is plotted. The data are representative of two biological replicates. There were no significant differences in viability between WT POLRMT and any POLRMT modification mimics (Student’s *t*-test, *p* > 0.1). (**B**) Relative mtDNA content of cells transfected with WT, T315E, or T315A POLRMT. DNA was isolated 48 h after transfection. Relative mtDNA content was measured using qPCR with gene-specific primers for a region in the nuclear genome (*18S*) and mitochondrial genome (*COX3*). Data are presented relative to the overexpression of WT POLRMT and represent the average and standard deviation of two experiments and six technical replicates. Results are representative of two biological replicates. No significant differences were observed (n.s. indicates no significance, *p* > 0.2, Student’s *t*-test). (**C**) Relative transcript level of eight mitochondrial encoded genes. The overexpression of T315E and T315A POLRM appears to increase the mitochondrial transcript level for the mitochondrial genes selected. Transcript levels were measured using qPCR with gene-specific primers using a TaqMan^®^ custom array. Transcript levels were determined using the ΔΔC_t_ method and normalized to *18S*. Transcript levels are presented relative to the overexpression of WT POLRMT. The average of three technical replicates is plotted. Error bars represent the upper and lower bounds of expression. Gene selection represents areas from across the mitochondrial genome. Abbreviations: *18S*: 18S ribosomal RNA; *ND1*, *ND5*, *ND6*: NADH dehydrogenase subunits 1, 5, 6; *CYB*: Cytochrome B; *CO1*, *CO3*: cytochrome c oxidase 1, 3; *ATP8*: ATP synthase F0 subunit 8; *RNR1*: mitochondrial encoded 12S rRNA.

To determine whether modification of POLRMT alters mitochondrial genome maintenance and expression in cells, we overexpressed POLRMT modification mimics (K402Q, T315E, T993E) and controls (K402R, T315A, T993A) in HeLa cells. The lysine to arginine mutant and threonine to alanine mutant serve as negative controls. Arginine mimics the size and shape of lysine but is unable to undergo acetylation. Alanine is unable to be phosphorylated. POLRMT overexpression was confirmed by qPCR and western blotting. Cell viability was measured 48 h after transcription using an alamarBlue^TM^ assay, as loss in viability may suggest a defect in mitochondrial respiration due to changes in POLRMT function. There were no significant changes in viability between cells overexpressing WT POLRMT and the modification mimics (Figure 3A). 

We focused the remainder of our cellular analysis on the effect of the T315E phosphomimic, as this was the only site to display any changes in promoter binding in vitro (Figure 2). DNA and RNA were isolated 48 h after transfection with WT POLRMT, T315E POLRMT, or T315A POLRMT. qPCR was used to analyze mtDNA content from transfected cells by determining the ratio of expression of a mitochondrial encoded gene (COXIII) to a nuclear encoded gene (18S) [28]. mtDNA content values were compared to cells overexpressing WT POLRMT (Figure 3B). The overexpression of T315E POLRMT had no effect on mtDNA content, suggesting that phosphorylation at this site does not impact mtDNA replication or genome maintenance. We also analyzed mtDNA content for the other mimics. We found that modification at T993 had no effect on mtDNA content. The acetyl mimic at K402 resulted in a slight increase in mtDNA, although these differences were not statistically significant across biological replicates (Supplemental Figure S1). 

Finally, we used qPCR to monitor changes in mtDNA transcription for eight mitochondrial transcripts using a custom commercial TaqMan^TM^ array plate. Transcript levels were compared to the overexpression of WT POLRMT (Figure 3C). Generally, there is an increase in transcript level upon overexpression of both the T315E and T315A mutants when compared to WT POLRMT. However, the magnitude of change between the T315E (phosphomimic) and T315A (dephosphorylated control) is minimal and within the upper and lower bounds of the expression ranges for nearly every gene. If phosphorylation at T315 is having an impact on mitochondrial transcription, we would expect changes in transcription upon the overexpression of T315E, with minimal changes in transcription upon overexpression of the T315A control. These results suggest that structural alterations at T315 may change POLRMT activity in cells, but this may not be a direct result of phosphorylation.

## 3. Discussion

LC-MS/MS proteomic analyses show that POLRMT is subject to phosphorylation and acetylation at numerous sites. Using protein point mutations that mimic lysine acetylation and threonine phosphorylation, we studied the effect of three modifications in structurally distinct regions on POLRMT function. Fluorescence anisotropy revealed no significant difference in POLRMT light strand promotor binding in vitro for two of the three mimics. A small decrease in K_d_ of approximately 2 nM was observed for the phosphomimic at threonine 315. While this finding reached statistical significance, our follow-up studies in HeLa cells did not reveal changes in cell viability, mtDNA content, or mitochondrial transcript level upon overexpression of the T315E POLRMT phosphomimic. Taken together, these results suggest that point mutants of POLRMT that mimic PTM at the sites studied do not significantly alter the ability of POLRMT to transcribe mtDNA or synthesize primers required for mtDNA replication. An important consideration when interpreting these results is that amino acid point mutants (K→Q, T→E) are not perfect representations of the modified forms of the amino acids. Therefore, it is still possible that PTMs do alter the parameters investigated, but the actual modification is required. It is also possible that other modification sites on POLRMT that were not studied here may change POLRMT function, or that multiple sites must be modified simultaneously to have a functional impact. 

Previous experiments investigating the effect of PTMs on another mitochondrial transcription factor, TFAM, found similar results to those presented here. While protein kinase A and acetyl-CoA were found to phosphorylate and acetylate TFAM, respectively, neither modification significantly impacted TFAM’s ability to compact mtDNA or initiate transcription [29]. However, modified TFAM appeared to slightly promote processivity of transcription by acting as less of a “roadblock” for POLRMT. The authors suggested that this subtle regulatory effect may be an evolutionary derived character of TFAM that prevents its function from being drastically affected by spurious acetylation in the acetyl-CoA rich matrix. POLRMT, which also resides in the matrix, may have evolved to exhibit an analogous response to acetylation. Lysine acetylation of mitochondrial proteins is widespread, with estimates of over 60% of the mouse mitochondrial proteome containing at least one acetyl site [30]. However, many of these sites occur at low levels, with stoichiometry measurements suggesting that the average level of acetylation is approximately 0–5% of the total protein amount [31]. As our ability to detect PTM sites on proteins increases with sensitive mass spectrometry approaches, it is possible that we identified lowly abundant acetyl sites on POLRMT with little impact on biological function. 

A similar line of reasoning may explain the inconsequential effect of phosphorylation. Protein phosphorylation is abundant in the mitochondrial proteome. It is estimated that nearly 91% of mitochondrial proteins contain a phosphorylation site, with an average of eight distinct phosphorylation sites per protein [10]. These phosphorylation events could also be of low stoichiometry and result in insignificant or subtle effects on protein function. The lack of dedicated mitochondrial protein kinases also raises the question of where mitochondrial proteins are being phosphorylated. In an experiment using a fluorescence resonance energy transfer (FRET)-based sensor to visualize cAMP-dependent kinase activity in the mitochondrial matrix and outer mitochondrial membrane, no protein kinase A activity was detected in the matrix, but high cAMP-dependent kinase activity was observed on the outer mitochondrial membrane [32]. Phosphorylation of nuclear-encoded mitochondrial proteins like POLRMT may occur upon transport across the outer mitochondrial membrane, and have minimal functional impact. While we do not completely understand where mitochondrial proteins are phosphorylated, there is evidence for robust phosphatase activity in the mitochondria [10]. However, POLRMT does not appear as one of the top potential substrates for the mitochondrial phosphatase PPTC7 [33]. This supports the idea that POLRMT phosphorylation may not be subject to regulation. Further experiments that investigate mtDNA transcription via alternative or more sensitive methods than those considered in this study may help to identify more subtle effects of PTMs on POLRMT function.

## 4. Materials and Methods

### 4.1. Mass Spectrometry

#### 4.1.1. Cell Culture, Transfection

HeLa cells were obtained from ATCC^®^ and were cultured in Eagle’s Minimum Essential Media (EMEM, ATCC^®^, Manassas, VA, USA) supplemented with 10% fetal bovine serum in a 37 °C, 5% CO_2_ humidified incubator using standard tissue culture protocols. Vectors expressing POLRMT or POLRMT modification mimics with C-terminal FLAG tags were purchased from Vector Builder Inc. (Chicago, IL, USA). Plasmids were transfected using Promega FuGENE^TM^6 (Fitchburg, WI, USA) following the manufacturer’s protocol. 

#### 4.1.2. Immunoprecipitation and Mass Spectrometry Sample Preparation

The immunoprecipitation procedure was adapted from Floyd et al. [34]. Transfected HeLa cells were harvested in lysis buffer (25 mM Tris pH 7.4, 100 mM NaCl, 0.5% NP-40, 0.5% Triton X-100, containing protease, phosphatase, and deacetylase inhibitor cocktails). Cells were lysed by sonication. The cell lysate was transferred to Pierce^TM^ anti-DYKDDDDK magnetic beads (Waltham, MA, USA). Samples were incubated overnight at 4 °C. Three washes were performed with wash buffer (25 mM Tris pH 7.4, 100 mM NaCl). POLRMT was eluted using 1.6 µg of 3xFLAG-peptide. Eluted samples were combined in a 1:1 ratio with 12 M urea in 0.2 M NH_4_HCO_3_. A total of 5 mM dithiothreitol (DTT) was added to samples and incubated for 30 min at 56 °C with gentle shaking. A total of 20 mM iodoacetamide (IAA) was added to samples and incubated in the dark for 20 min. DTT was added to a final concentration of 10 mM, and 0.2 M NH_4_HCO_3_ was added until the concentration of urea was below 2 M. A total of 1 µg of trypsin or Glu-C (Promega, Fitchburg, WI, USA) was added to samples and incubated at 37 °C overnight with gentle shaking. Trifluoroacetic acid (TFA) was used to quench the reaction and samples were dried using a speed vacuum. Samples were resuspended in 100 µL of 0.1% TFA and then desalted using Pierce^TM^ C18 ZipTips (Waltham, MA, USA). Samples were dried using a speed vacuum, and peptides were resuspended in 20 µL of 5% acetonitrile/0.1% formic acid. Samples were analyzed using an Agilent 6545 ESI Q-TOF (Santa Clara, CA, USA). Using an iterative injection method, three 6 µL injections were automatically injected by an Agilent 1260 Infinity II (Santa Clara, CA, USA) onto a C18 column (Poroshell 120, EC-C18, 3.0 × 100 mm, 2.7 μm) maintained at 60 °C. Peptides were eluted over a 35 min gradient at a flow rate of 0.4 mL/min (5–30% B for 5 min, 30–90% B for 27 min, 90% B hold; Buffer A = 99.9% H_2_O/0.1% formic acid; Buffer B = 5% acetonitrile/0.1% formic acid/94.9% H_2_O). Eluted peptides were sprayed into an Agilent 6545 LC/Q-TOF spectrometer using a dual AJS ESI (Agilent Jet Stream Electrospray Ionization) source (Agilent, Santa Clara, CA, USA). The top 3 ions in each survey scan cycle were subjected to collision-induced dissociation (using the formula: collision energy = 3.6 × *m*/*z*/100 − 4.8). Fragment spectra were acquired at a rate of 5 spectra/second and a threshold of 2000 counts. Two reference mass ions were continually flowed through the MS during data acquisition.

#### 4.1.3. Data Analysis

Spectra were analyzed using BioConfirm 10.0 (Agilent, Santa Clara, CA, USA). BioConfirm was used to search for POLRMT peptides and post-translational modifications. MS/MS scores of matched peptides above 50 were determined to be authentic, if accompanied by appropriate chromatograph traces and b and y ions. Proteomic data, including raw data files and Bioconfirm result files were submitted to ProteomeXchange (doi:10.25345/C57P8TQ4W, data set identifier: PXD046430). Supplementary Table S1 contains POLRMT peptide identification results. 

### 4.2. Site-Directed Mutagenesis and Protein Purification

The bacterial expression vector for POLRMT (pProEX HtB) was a kind gift from Dr. Smita Patel [27]. Site-directed mutagenesis was performed to create mimics of post-translationally modified proteins in both the bacterial and mammalian expression vector using the Agilent QuikChange Lightning system (Santa Clara, CA, USA) according to the manufacturer’s protocol. The following primers were used for mutagenesis: T315E Forward: 5′-TTGTCCAGACAACGTTCAATCTCACCGGCGTCCTGATCTTGAC-3′; T315E Reverse: 5′-GTCAAGATCAGGACGCCGGTGAGATTGAACGTTGTCTGGACAA-3′; T315A Forward: 5′-ACAACGTTCAATGGCACCGGCGTCCTGATCTTG-3′; T315A Reverse: 5′-CAAGATCAGGACGCCGGTGCCATTGAACGTTGT-3′; K402Q Forward: 5′-CCATGTGGAGCTGCTGCTCAAAGAGGCACTG-3′; K402Q Reverse: 5′CAGTGCCTCTTTGAGCAGCAGCTCCACATGG-3′; K402R Forward: 5′-GTGGAGCTGCCTCTCAAAGAGGCACTGCAG-3′; K402R Reverse: 5′-CTGCAGTGCCTCTTTGAGAGGCAGCTCCAC-3′; T993E Forward: 5′-ACCATAGACAACCGTCATAACTTCCTGTTTCACGACTTTGCGCGT-3′; T993E Reverse: 5′-ACGCGCAAAGTCGTGAAACAGGAAGTTATGACGGTTGTCTATGGT-3′; T993A Forward: 5′-GACAACCGTCATAACGGCCTGTTTCACGACTTTGC-3′; T993A Reverse: 5′-GCAAAGTCGTGAAACAGGCCGTTATGACGGTTGTC-3′. 

POLRMT was purified as described previously [27]. Plasmids obtained by site-directed mutagenesis were transformed into BL21 *E. coli* cells. Starter cultures were grown in 2XYT overnight and used to inoculate 500 mL cultures. Cultures were grown to an OD600 of 0.6. Protein expression was induced by adding 0.5 mM IPTG, and cultures were incubated for 16 h at 16 °C. Cells were isolated by centrifugation and lysed chemically (lysis buffer: 40 mM Tris-HCl, 300 mM NaCl, 15% glycerol, 0.1% Tween-20, 1 mM PMSF, 1 mM EDTA, 0.05 mM DTT, 1 mg/mL lysozyme, 5 μg/mL aprotinin, 5 μg/mL leupeptin) and by sonication. Polyethyleneimine precipitation was used to remove nucleic acids, and 55% saturation of ammonium sulfate was used to precipitate the protein. Then, a medium pressure liquid chromatography system was used to perform DEAE anion exchange column chromatography and nickel affinity column chromatography to purify POLRMT (DEAE buffer: 40 mM Tris-HCl, 300 mM NaCl, 15% glycerol, 0.1% Tween-20, 1 mM PMSF, 0.3 mM EDTA, 0.05 mM DTT, and 20 mM imidazole followed by 500 mM imidazole). A heparin column was used to further purify the protein and remove residual DNA (heparin buffer: 40 mM Tris-HCl, 15% glycerol, 0.1% Tween-20, 1 mM PMSF, 0.3 mM EDTA, 0.05 mM DTT, 20 mM imidazole, and 150 mM NaCl followed by 1 M NaCl). After purification, the protein was concentrated using Corning^®^ Spin-X UF concentrators (Salt Lake City, UT, USA) in a tabletop centrifuge and dialyzed in dialysis buffer (50 mM Tris-HCl, 150 mM NaCl, 50% glycerol, 0.1% Tween-20, 1 mM EDTA, and 1 mM DTT). 

### 4.3. Fluorescence Anisotropy

Fluorescence polarization measurements were performed as previously described [27]. A 20 mM Tris-HCl EDTA buffer, nuclease-free water, and 3 nM fluorescein-labeled duplex oligos (light strand promoter region −40 to +10) were pipetted evenly into a black 96-well plate. Wild-type (WT) POLRMT or PTM mimic was titrated from 0–20 nM. The plate was read on an Agilent Synergy H1 microplate reader (Agilent, Santa Clara, CA, USA) at 25 °C with a read height of 7.00 mm and one minute of linear shaking prior to reading. The polarization filters used were 485/20, 528/20 [parallel], and 485/20, 528/20 [perpendicular]. Anisotropy values were recorded for each well of the plate. The dissociation constants (K_d_) of POLRMT-DNA complexes were determined using a nonlinear regression for specific binding with the Hill slope. Data were analyzed in GraphPad Prism version 9.5.1.

### 4.4. Viability Measurements

Cell viability was determined 48 h post transfection with POLRMT modification mimics using alamarBlue^TM^ (Waltham, MA, USA) following the manufacturer’s protocol. 

### 4.5. Mitochondrial DNA Content Assay

mtDNA copy number was measured by SYBR Green fluorescence [24]. DNA was extracted from cells using the Zymo© Quick-DNA kit (Irvine, CA, USA) according to the manufacturer’s protocol 48 h post transfection. Total DNA samples were diluted to 1 ng/μL and combined with Bio-Rad© SYBR Green master mix (Hercules, CA, USA) and 0.625 µM forward and reverse primers. Primers amplifying the 18S region of nuclear DNA (For: 5′-TAGAGGGACAAGTGGCGTTC-3′; Rev: 5′-CGCTGAGCCAGTCAGTGT-3′) and COX3 region (For: 5′-CACCCAAGAACAGGGTTTGT-3′; Rev: 5′-TGGCCATGGGTATGTTGTTAA-3′) of mtDNA were used. The difference between the mitochondrial and nuclear C_t_ values was determined and used to calculate the relative mtDNA copy number. 

### 4.6. qPCR

RNA was isolated from transfected cells using the Zymo© Quick-RNA kit (Irvine, CA, USA) according to the manufacturer’s protocol 48 h post transfection. RNA was quantified and converted to cDNA using the Bio-Rad© iScript cDNA synthesis kit (Hercules, CA, USA). SYBR Green-based qPCR (Actin B: IDT 401607592 RxnReady Primer Pool; POLRMT: For: 5′-AGAAGCGGAAGCTGCTCA-3′; Rev: 5′-GGCATCCTTCACCATGAATAA-3′) or custom TaqMan^TM^ gene expression arrays (18S-Hs99999901_s1: eukaryotic 18S rRNA; MT-ND1-Hs02596873_s1: NADH dehydrogenase 1; MT-ND5-Hs02596878_g1: NADH dehydrogenase 5; MT-ND6-Hs02596879_g1: NADH dehydrogenase 6; MT-CYB-Hs02596867_s1: cytochrome b; MT-CO1-Hs02596864_g1: cytochrome c oxidase I; MT-CO3-Hs02596866_g1: cytochrome c oxidase III; MT-ATP8-Hs02596863_g1: ATP synthase 8; MT-RNR1-Hs02596859_g1: 12S RNA; ACTB-Hs99999903_m1: actin beta) were used to confirm overexpression and determine transcript levels, respectively. For SYBR Green-based analysis, the manufacturer’s PCR protocol (PowerUp SYBR Green Master Mix, Applied Biosystems, Waltham, MA, USA) was followed. A constant annealing temperature of 60 °C was used for all primers, and a melt curve was performed. For TaqMan^TM^ analysis, the manufacturer PCR protocol was followed (TaqMan^TM^ Fast Advanced Master Mix for qPCR, Applied Biosystems, Waltham, MA, USA) with a constant annealing temperature of 60 °C for all primer mixes. The ΔΔC_t_ method was used for analysis, with the nuclear encoded 18S gene serving as the control. The upper and lower limits of expression were calculated from three technical replicates.

### 4.7. Statistical Analysis

Statistical Package for Social Sciences (IBM SPSS) version 28.0.0.0 or GraphPad Prism version 9.5.1 were used to perform *t*-tests to assess significance. A *p*-value of 0.05 or less was considered statistically significant. Details of statistical analysis are presented in figure legends where applicable. 

## Data Availability

The data presented in this study are available in this article or upon request. Proteomic spectral reports are provided in the Supplementary Materials.

## Supplementary Materials

The supporting information can be downloaded at https://www.mdpi.com/article/10.3390/ijms242216050/s1.
