# Supplementary material for: Assessing the Role of Post-Translational Modifications of Mitochondrial RNA Polymerase
Source: Int J Mol Sci. 2023 Nov 7;24(22):16050. doi: 10.3390/ijms242216050 (PMC10671485; doi:10.3390/ijms242216050)
Supplement: Supplementary file 1 [file ijms-24-16050-s001.zip › ijms-2683037-Figure S1.pdf]

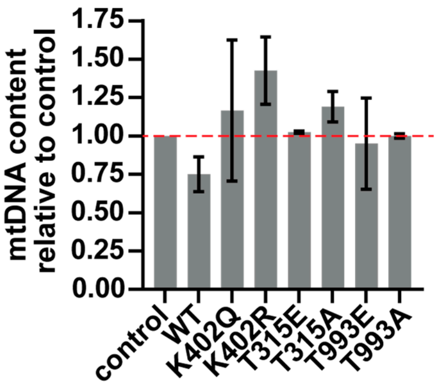

Supplemental Figure S1. mtDNA content of POLRMT modification mimics. Relative mtDNA content of modification mimics compared to an un-transfected control. Relative mtDNA was measured using qPCR with gene-specific primers for a region in the nuclear genome (18S) and mitochondrial genome (COX3). Data represent the average and standard deviation of two experiments and four technical replicates, and are representative of multiple biological replicates. There are no significant differences in mtDNA content between transfected cells and the untransfected control (Student's *t*-test, $p>0.1$).
